# Multiple-Parameter Estimation Method Based on Spatio-Temporal 2-D Processing for Bistatic MIMO Radar

**DOI:** 10.3390/s151229865

**Published:** 2015-12-14

**Authors:** Shouguo Yang, Yong Li, Kunhui Zhang, Weiping Tang

**Affiliations:** 1 School of Electronics and Information, Northwestern Polytechnical University, Xi’an 710072, China; ruikel@nwpu.edu.cn (Y.L.); kunhuizhang@hotmail.com (K.Z.); 2 Air and Missile Defense College, Air Force Engineering University, Xi’an 710051, China; twp716@163.com

**Keywords:** spatio-temporal 2-D processing, bistatic MIMO radar, transmitting-receiving azimuth, Doppler frequency

## Abstract

A novel spatio-temporal 2-dimensional (2-D) processing method that can jointly estimate the transmitting-receiving azimuth and Doppler frequency for bistatic multiple-input multiple-output (MIMO) radar in the presence of spatial colored noise and an unknown number of targets is proposed. In the temporal domain, the cross-correlation of the matched filters’ outputs for different time-delay sampling is used to eliminate the spatial colored noise. In the spatial domain, the proposed method uses a diagonal loading method and subspace theory to estimate the direction of departure (DOD) and direction of arrival (DOA), and the Doppler frequency can then be accurately estimated through the estimation of the DOD and DOA. By skipping target number estimation and the eigenvalue decomposition (EVD) of the data covariance matrix estimation and only requiring a one-dimensional search, the proposed method achieves low computational complexity. Furthermore, the proposed method is suitable for bistatic MIMO radar with an arbitrary transmitted and received geometrical configuration. The correction and efficiency of the proposed method are verified by computer simulation results.

## 1. Introduction

In recent years, substantial research on target location for bistatic multiple-input multiple-output (MIMO) radar has been conducted by many scholars [1,2,3,4], mainly on the joint estimation of the target transmitting-receiving azimuth [5,6,7]. However, the echo signal of bistatic MIMO radar includes not only direction of departure (DOD) and direction of arrival (DOA) but also Doppler frequency information on the target. DOD and DOA can be estimated based on the received echo signal; moreover, the Doppler frequency can also be estimated. Thus, the cross localization of the target and the joint estimation of speed can be realized.

Many different algorithms, mainly addressing the above questions, have been presented in the literature. In reference [8], a joint estimation algorithm concerning the transmitting-receiving azimuth and Doppler frequency of bistatic MIMO radar is proposed based on the method of ESPRIT. The rotational invariance factor can be obtained using the sampling delay. The estimated angle and Doppler frequency can be matched automatically, and the loss of array aperture can be avoided. In reference [9], the bistatic MIMO radar receiver signal expression of a moving target is derived under the Sweiling II target model, which provides three-way array model characteristics; moreover, a joint estimation algorithm is proposed based on PARAFAC. In reference [10], a reasonable cost function is constructed using the biorthogonality of matrices. By iteratively solving the cost function and using a systematic multistage decomposition process, the transmitting-receiving azimuth and Doppler frequency of each target are estimated in turn. In references [11,12], a multi-target signal model for bistatic MIMO radar is established, and a joint angles-Doppler frequency estimation algorithm is proposed based on the method of propagation factors. However, most of the above algorithms are only suitable for applications considering spatial white Gaussian noise and a known number of targets. In the presence of spatial colored noise and an unknown number of targets, the performance of parameter estimation can be greatly reduced or fully compromised. Focusing on these problems, a novel spatio-temporal 2-dimensional (2-D) processing method that can jointly estimate the transmitting-receiving azimuth and Doppler frequency for bistatic MIMO radar is proposed.

The organization of the paper is as follows: the data model of a bistatic MIMO radar system is presented in Section 2. In Section 3, a novel spatio-temporal 2-D processing method that can jointly estimate the transmitting-receiving azimuth and Doppler frequency for bistatic MIMO radar is proposed in the presence of spatial colored noise and an unknown number of targets. In Section 4, three computer simulation results of target parameters generated by the algorithm in the method proposed here and in other references are presented. Finally, Section 5 concludes the paper.

***Notation*:** The operators ⊗, ⊙ and ∘ denote the Kronecker product, Hadamard product and Khatri-Rao product, respectively; *diag*(·) denotes a diagonal matrix composed of the columns or row vectors; vec(⋅) represents the matrix vectorization by column; *angle*(·) denotes the phase of a plural; *E*[·] denotes the expected value; ${\left\|\cdot \right\|}_{\text{2}}$ denotes the $\ell_{2}$ norm; (·)^*^, (·)*^T^*, (·)*^H^,* (·)*^–1^* and (·)*^#^* represent the complex conjugate, transpose, Hermitian transpose, matrix inverse and pseudo inverse, respectively; *Ia* is an *a* × *a* identity matrix, a is a positive integer.

## 2. Data Model

Considering a bistatic MIMO radar system in which the transmitting and receiving arrays are all linear arrays, the number of transmitting array elements is $M_{t}$. Each of the transmitting array elements emits the same frequency and orthogonal phase code signal at the same time, and the number of receiving array elements is *M_r_*. Suppose that the baseline distance between the transmitting and receiving arrays is *D*, where D≫λ, and λ is the wavelength of the carrier. It is also assumed that there are *P* targets at the same distance unit in the far field of the radar system and that the 2-D azimuth relative to the transmitting and receiving arrays is $\left(\varphi_{p},\theta_{p}\right),p=1,2,\cdots ,P$. Thus, in the case of a point target, the echo signal received by the bistatic MIMO radar in the *l*th pulse cycle (l=1,2,⋯,L) can be expressed as:

(1) $$X(t_{l})={\left[x_{1},x_{2},\cdots x_{M_{r}}\right]}^{T}=\sum_{p=1}^{P}\xi_{p}a_{r}\left(\theta_{p}\right){a_{t}}^{T}\left(\varphi_{p}\right)Se^{j2\pi f_{\;dp}t_{l}}+W\left(t_{l}\right),l=1,2,\cdots L$$

where $x_{n}$ is the echo signal of the *n*th receiving array element, $\xi_{p}$ is the reflection coefficient of the *p*th target, $f_{dp}$ is the normalization Doppler frequency of the *p*th target, $a_{r}(\theta_{p})$ is the steering vector in the receiving array according to the *p*th target, $a_{t}(\varphi_{p})$ is the steering vector in the transmitting array according to the *p*th target, $S={\left[s_{1},s_{2},\cdots s_{M_{t}}\right]}^{T}$, $s_{m}$ is the orthogonal signal emitted by the *m*th transmitting array element, and $W\left(t_{l}\right)\in {\mathbb{C}}^{M_{r}\times K}$ is Gaussian noise with a mean value of 0.

After the echo signal is match-filtered and stacked according to column, Equation (1) can be expressed as follows [6,7]:

(2) $$y\left(t_{l}\right)=A\beta \left(t_{l}\right)+n\left(t_{l}\right),\;l=1,2,\cdots ,L$$

where:

$$A=[a(\varphi_{1},\theta_{1}),a(\varphi_{2},\theta_{2}),\cdots ,a(\varphi_{P},\theta_{P})]$$

$$a(\varphi_{p},\theta_{p})=a_{r}(\theta_{p})⊗a_{t}(\varphi_{p})$$

$$\beta (t_{l})=diag\left\{\left[\xi_{1}e^{j2\pi f_{d1}t_{l}},\xi_{2}e^{j2\pi f_{d2}t_{l}},\cdots ,\xi_{p}e^{j2\pi f_{dP}t_{l}}\right]\right\}$$

and $n(t_{l})=vec\left(W(t_{l})S^{H}\right)$ is the virtual noise of the entire bistatic MIMO radar system after matched filter, which is the Gaussian noise with a mean value of 0.

Suppose that the array additive noise $W\left(t_{l}\right)$ in Equation (1) is Gaussian white noise in time domain and Gaussian noise with a mean value of 0 in spatial domain, each column of $W\left(t_{l}\right)$ is an independent Gaussian noise vector with identical distributions, for which the covariance matrix is unknown and defined as $Q_{w}$.

Thus, we can obtain [13]:

(3) $$E\left\{vec\left[W\left(t_{i}\right)\right]vec^{H}\left[W\left(t_{j}\right)\right]\right\}=\left\{ \begin{array}{ll} I_{k}⊗Q_{w}, & i=j\\ 0, & i\neq j \end{array}\right.$$

Therefore, the correlation matrix of the noise vector exported by matched filters for the *i*th and *j*th pulse cycle can be expressed as:

(4) $$\begin{array}{l} E\left[n\left(i\right)n^{H}\left(j\right)\right]=E\left\{vec\left[W\left(t_{i}\right)S^{H}\right]vec^{H}\left[W\left(t_{j}\right)S^{H}\right]\right\}\\ \begin{array}{cc} = & E\left\{\left[S^{*}⊗I_{M_{r}}\right]\left[vec(W\left(t_{i}\right))vec^{H}(W\left(t_{i}\right))\right]\left[S^{T}⊗I_{M_{r}}\right]\right\} \end{array}\\ \begin{array}{c} \begin{array}{c} =\left\{ \begin{array}{ll} \left[S^{*}⊗I_{M_{r}}\right]\left[I_{k}⊗Q_{w}\right]\left[S^{T}⊗I_{M_{r}}\right], & i=j\\ 0, & i\neq j \end{array}\right. \end{array} \end{array}\\ \begin{array}{cc} = & \left\{ \begin{array}{ll} I_{Mt}⊗Q_{w}, & i=j\\ 0, & i\neq j \end{array}\right. \end{array} \end{array}$$

The above formula shows that the noise item is 0 after the outputs of matched filters for different pulse cycle have been cross-correlated.

## 3. Multiple-Parameter Estimation Spatio-Temporal 2-D Processing Method

### 3.1. Estimation of Transmitting-Receiving Azimuth

According to Equation (2), we can construct three $M_{t}M_{r}\times \left(L-2\right)$-dimensional data matrixes with the outputs of matched filters for L pulse cycle, they are as follows:

(5) $$Y_{1}=\left[y\left(t_{1}\right),y\left(t_{2}\right),\cdots ,y\left(t_{L-2}\right)\right]=A\beta_{1}+N_{1}$$

(6) $$Y_{2}=\left[y\left(t_{2}\right),y\left(t_{3}\right),\cdots ,y\left(t_{L-1}\right)\right]=A\beta_{2}+N_{2}$$

(7) $$Y_{3}=\left[y\left(t_{3}\right),y\left(t_{4}\right),\cdots ,y\left(t_{L}\right)\right]=A\beta_{3}+N_{3}$$

where $\beta_{1}=\left[\beta \left(t_{1}\right),\beta \left(t_{2}\right),\cdots ,\beta \left(t_{L-2}\right)\right]$, $\beta_{2}=\left[\beta \left(t_{2}\right),\beta \left(t_{3}\right),\cdots ,\beta \left(t_{L-1}\right)\right]$, $\beta_{3}=\left[\beta \left(t_{3}\right),\beta \left(t_{4}\right),\cdots ,\beta \left(t_{L}\right)\right]$, $N_{1}=\left[n\left(t_{1}\right),n\left(t_{2}\right),\cdots ,n\left(t_{L-2}\right)\right]$, $N_{2}=\left[n\left(t_{2}\right),n\left(t_{3}\right),\cdots ,n\left(t_{L-1}\right)\right]$, $N_{3}=\left[n\left(t_{3}\right),n\left(t_{4}\right),\cdots ,n\left(t_{L}\right)\right]$.

Because $\beta \left(t_{l}\right)=\xi_{p}e^{j2\pi f_{dp}t_{l}},l=1,2,\cdots L$, $t_{l}=\left(l-1\right)T_{r}$, where $T_{r}$ is the repetitive cycle of the pulse. Thus:

(8) $$\beta_{2}=\Phi_{f}\beta_{1}$$

(9) $$\beta_{3}=\Phi_{f}^{2}\beta_{1}$$

Here, $\Phi_{f}=diag\left[e^{j2\pi f_{d1}T_{r}},e^{j2\pi f_{d2}T_{r}},\cdots ,e^{j2\pi f_{dP}T_{r}}\right]$.

According to Equation (4), the noise matrixes $N_{1}$, $N_{2}$ and $N_{3}$ satisfy the following relationships:

(10) $$N_{2}N_{1}^{H}/\left(L-2\right)=0$$

(11) $$N_{3}N_{1}^{H}/\left(L-2\right)=0$$

Therefore, the cross-covariance matrixes of $Y_{2},Y_{3}$ and $Y_{1}$ are:

(12) $$R_{Y_{2}Y_{1}}=Y_{2}Y_{1}^{H}/\left(L-2\right)=A\Phi_{f}R_{\beta_{1}}A^{H}$$

(13) $$R_{Y_{3}Y_{1}}=Y_{3}Y_{1}^{H}/\left(L-2\right)=A\Phi_{f}^{2}R_{\beta_{1}}A^{H}$$

where $R_{\beta_{1}}=\beta_{1}\beta_{1}^{H}/\left(L-2\right)$. The two formulas above show that the proposed method eliminates the effect of spatial colored noise because the time sampling information is used reasonably.

By eigenvalue decomposition (EVD) of $R_{Y_{2}Y_{1}}$, we can get:

(14) $$R_{Y_{2}Y_{1}}=\left[\begin{array}{cc} V_{s} & V_{n} \end{array}\right]\left[\begin{array}{cc} \begin{array}{l} \Sigma_{s}\\ 0 \end{array} & \begin{array}{l} 0\\ 0\times I_{M_{t}M_{r}-P} \end{array} \end{array}\right]\left[\begin{array}{l} V_{s}^{H}\\ V_{n}^{H} \end{array}\right]=V_{s}\Sigma_{s}V_{s}^{H}+0\times V_{n}V_{n}^{H}$$

where $V_{s}=\left[v_{1},v_{2},\cdots v_{P}\right]$ and $V_{n}=\left[v_{P+1},v_{P+2},\cdots ,v_{M_{t}M_{r}}\right]$ are the signal subspace and noise subspace, respectively. $\Sigma_{s}=diag\left[\eta_{1},\eta_{2},\cdots ,\eta_{P}\right]$ is a diagonal matrix composed of the *P* non-zero eigenvalues.

To get the noise subspace, $R_{Y_{2}Y_{1}}$ is made to unit-diagonal-load using the diagonal-load method because the number of targets is unknown. That is:

(15) $$R_{DL}=R_{Y_{2}Y_{1}}+I_{M_{t}M_{r}}=V_{s}\left(\Sigma_{s}+I_{p}\right)V_{s}^{H}+V_{n}V_{n}^{H}$$

$I_{M_{t}M_{r}}=V_{s}V_{s}^{H}+V_{n}V_{n}^{H}$ is used in the derivation of the above formula.

Therefore:

(16) $$R_{DL}^{-m}=V_{n}V_{n}^{H}+V_{s}\left[\begin{array}{ccc} {\left(\frac{1}{\eta_{1}+1}\right)}^{m} & \cdots & 0\\ \vdots & \ddots & \vdots \\ 0 & \cdots & {\left(\frac{1}{\eta_{p}+1}\right)}^{m} \end{array}\right]V_{s}^{H}$$

where m is an arbitrary integer. Because $1/\left(\eta_{p}+1\right)$ is a number less than 1, Equation (16) approaches the noise subspace when m approaches infinite. *i.e*.,:

(17) $$\underset{m\to \infty }{\lim}R_{DL}^{-m}=V_{n}V_{n}^{H}$$

Thus, the noise subspace can be acquired without EVD of $R_{Y_{2}Y_{1}}$ and prediction of the number of targets. Equation (17) indicates that $R_{DL}^{-m}$ can converge to the noise subspace when m→∞. In fact, better performance can be acquired as long as m is a smaller integer.

Accordingly, the optimization equation for the estimation of the target transmitting-receiving azimuth can be obtained based on the subspace theory:

(18) $$\left[{\overset{\wedge }{\varphi }}_{p},{\overset{\wedge }{\theta }}_{p}\right]=\arg\underset{\varphi ,\theta }{\min}\left[a^{H}\left(\varphi ,\theta \right)R_{DL}^{-m}a\left(\varphi ,\theta \right)\right],\begin{array}{c} p=1,2,\cdots ,P \end{array}$$

From above, the judgment of the number of signal sources and the EVD of the data covariance matrix are not required in the estimation process of the algorithm. Therefore, this algorithm can greatly reduce the arithmetic complexity of the system.

Because $a\left(\varphi ,\theta \right)=a_{r}\left(\theta \right)⊗a_{t}\left(\varphi \right)$, according to the property of the Kronecker product, a(φ,θ) can be further expressed as follows:

(19) $$a\left(\varphi ,\theta \right)=\left[a_{r}\left(\theta \right)⊗I_{M_{t}}\right]a_{t}\left(\varphi \right)$$

Substituting Equation (19) into Equation (18), we can obtain the following:

(20) $$\left[{\overset{\wedge }{\varphi }}_{p},{\overset{\wedge }{\theta }}_{p}\right]=\arg\underset{\varphi ,\theta }{\min}\left[a_{t}^{H}\left(\varphi \right)F\left(\theta \right)a_{t}\left(\varphi \right)\right]$$

(21) $$F\left(\theta \right)={\left[a_{r}\left(\theta \right)⊗I_{M_{t}}\right]}^{H}{\hat{R}}_{DL}^{-m}\left[a_{r}\left(\theta \right)⊗I_{M_{t}}\right]$$

Because the first element of $a_{t}\left(\varphi \right)$ is 1, Equation (20) can be transformed into an optimization problem with constraints as follows:

(22) $$\left[{\overset{\wedge }{\varphi }}_{p},{\overset{\wedge }{\theta }}_{p}\right]=\arg\underset{\varphi ,\theta }{\min}\left[a_{t}^{H}\left(\varphi \right)F\left(\theta \right)a_{t}\left(\varphi \right)\right]\begin{array}{c} s.t.\;\begin{array}{c} e_{1}^{T}a_{t}\left(\varphi \right)=1 \end{array} \end{array}$$

where $e_{1}$ is the $M_{t}\times 1$-dimensional vector whose first element is 1 and other elements are 0.

By solving Equation (22), we can obtain the following:

(23) $${\hat{\theta }}_{p}=\arg\underset{\theta }{\min}\frac{1}{e_{1}^{T}F^{-1}\left(\theta \right)e_{1}}=\arg\underset{\theta }{\max}\left[e_{1}^{T}F^{-1}\left(\theta \right)e_{1}\right]$$

(24) $${\overset{\wedge }{a}}_{t}\left(\varphi_{p}\right)=\frac{F^{-1}\left({\hat{\theta }}_{p}\right)e_{1}}{e_{1}^{T}F^{-1}\left({\hat{\theta }}_{p}\right)e_{1}},\begin{array}{c} p=1.2.\cdots P \end{array}$$

According to Equation (23), searching for different θ in the range of $\left(-90^{\circ },90^{\circ }\right)$, we can obtain P maximal spectral peaks of the (1,1) element in $F^{-1}\left(\theta \right)$, which correspond to P DOA estimation values of the target. Then, substituting the P DOA estimation values obtained above into Equation (24), we can obtain the corresponding target transmit steering vector ${\overset{\wedge }{a}}_{t}\left(\varphi_{p}\right)$.

Suppose that $d_{t,m}$ is the distance between the *m*th transmitting array element and the reference array element. Given $d_{t,m}-d_{t,m-1}\leq \lambda /2,m=2,3,\cdots ,M_{t}$, ${\overset{\wedge }{\varphi }}_{p}$ can be calculated using the following formula:

(25) $${\hat{\varphi }}_{p}=\text{asin}\left[\frac{\lambda }{2\pi \left(M_{t}-1\right)}\sum_{m=2}^{M_{t}}\frac{\text{angle}\left({\hat{a}}_{tp,m}^{*}{\hat{a}}_{tp,m-1}\right)}{d_{t,m}-d_{t,m-1}}\right],\;p=1,2,\cdots ,P$$

where ${\hat{a}}_{tp,m}$ is the *m*th element of ${\hat{a}}_{t}\left({\hat{\varphi }}_{p}\right)$ and $d_{t,1}=0$.

Given that $d_{t,m}-d_{t,m-1}>\lambda /2,m=2,3,\cdots ,M_{t}$, the estimation of ${\hat{\phi }}_{p}$ may be wrong according to Equation (25) because the estimation of ${\hat{a}}_{tp,m}^{*}{\hat{a}}_{tp,m-1}$ may be ambiguous. Thus, ${\overset{\wedge }{\varphi }}_{p}$ can be acquired using a 1-dimensional search as follows:

(26) $${\hat{\varphi }}_{p}=\arg\underset{\varphi }{\max}\left|a_{t}^{H}\left(\varphi \right){\hat{a}}_{t}\left(\varphi_{p}\right)\right|,p=1,2,\cdots ,P$$

To estimate the transmit steering vector ${\hat{a}}_{t}\left(\varphi_{p}\right)$, the 1-dimensional search can be performed according to Equation (26) in the range of $\varphi \in \left(-90^{\circ },90^{\circ }\right)$. Its maximum value is the DOD estimated value ${\hat{\varphi }}_{p}$, which can be automatically matched with the estimated receiving azimuth.

### 3.2. Estimation of the Doppler Frequency

Suppose that $\lambda_{1},\lambda_{2},\cdots ,\lambda_{p}$ are P non-zero singular values of the matrix $R_{Y_{2}^{'}Y_{1}^{'}}$, $u_{1},u_{2},\cdots ,u_{P}$ and $v_{1},v_{2},\cdots ,v_{P}$ are, respectively, the left-singular vector and right-singular vector corresponding to the P non-zero singular values. The pseudo inverse of $R_{Y_{2}^{'}Y_{1}^{'}}$ is defined as follows:

(27) $$R_{Y_{2}^{\prime }Y_{1}^{\prime }}^{\#}≜\sum_{p=1}^{P}\frac{1}{\lambda_{p}}v_{p}u_{p}^{H}$$

Then, the matrix G can be constructed as:

(28) $$G≜R_{Y_{3}^{\prime }Y_{1}^{\prime }}R_{Y_{2}^{\prime }Y_{1}^{\prime }}^{\#}$$

Therefore, we can obtain:

(29) $$GA=A\Phi_{f}$$

Equation (29) means that $\Phi_{f}\left(p,p\right),p=1,2,\cdots ,P$ is the eigenvalue of the matrix G, and $a\left(\varphi_{p},\theta_{p}\right)$ is the corresponding eigenvector.

In fact, because the number of repetitive pulses is limited, Equation (29) is not strictly true. However, the 2-D azimuth $\left(\varphi_{p},\theta_{p}\right)$ of the target and the Doppler frequency $f_{dp}$ can be jointly estimated through the following optimization problem:

(30) $$\left[{\hat{\varphi }}_{p},{\hat{\theta }}_{p},{\hat{f}}_{dp}\right]=\arg\underset{\varphi ,\theta ,f_{d}}{\min}\|Ga\left(\varphi ,\theta \right)-{e^{j2\pi f_{d}T_{s}}a\left(\varphi ,\theta \right)\|}_{2},\;p=1,2,\cdots ,P$$

From Equation (30), the transmitting-receiving azimuth and Doppler frequency are separable. The 2-D azimuth $\left({\hat{\varphi }}_{p},{\hat{\theta }}_{p}\right)$ of the target can be obtained through the method proposed in Section 3.1. Meanwhile, given that the derivative of the target function of Equation (30) with respect $f_{d}$ is zero, the estimated Doppler frequency ${\hat{f}}_{dp}$ can be obtained by minimizing the target function:

(31) $${\hat{f}}_{dp}=\frac{\text{angle}\left[a^{H}\left({\hat{\varphi }}_{p},{\hat{\theta }}_{p}\right)Ga\left({\hat{\varphi }}_{p},{\hat{\theta }}_{p}\right)\right]}{2\pi T_{s}M_{t}M_{r}},\;p=1,2,\cdots ,P$$

Therefore, substituting the estimated transmitting-receiving azimuth into the formula above, we can obtain the target Doppler frequency, and the acquired target Doppler frequency can be automatically matched with the transmitting-receiving azimuth.

During parameter estimation, only the time rotation factor is used; the array rotation invariance is not used. No special requirement for the array structure is required in this method, which is applied to the condition of the arbitrary structure of the transmitting and receiving array. For the algorithm proposed in [13], when $d_{t,m}-d_{t,m-1}>\lambda /2,m=2,3,\cdots ,M_{t}$ and $d_{r,n}-d_{r,n-1}>\lambda /2,n=2,3,\cdots ,M_{r}$, an error in the angle estimation may occur, as is the case for Equation (25). Thus, it is only suitable for bistatic MIMO radars whereby $d_{t,m}-d_{t,m-1}\leq \lambda /2,m=2,3,\cdots ,M_{t}$ and $d_{r,n}-d_{r,n-1}\leq \lambda /2,n=2,3,\cdots ,M_{r}$. Therefore, the requirement for the distance of the transmitting and receiving array in the proposed method is lower than that of the algorithm proposed in reference [13], and this method is applicable over a wider range.

## 4. Computer Simulation Analysis

In the simulation process, the Signal-to-Noise Ratio (SNR) is 5 dB; m=2; the number of pulses is L, where L=100; $M_{t}=6,\begin{array}{c} M_{r}=8,\begin{array}{c} f_{s}=10 \end{array} \end{array}$ KHz. Suppose that there are three targets within the same distance unit in the background with Gaussian colored noise, the transmitting-receiving azimuth and the Doppler frequency are $\left(10^{\circ },20^{\circ },1000\;\text{Hz}\right)$, $\left(-8^{\circ },30^{\circ },2300\;\text{Hz}\right)$ and $\left(0^{\circ },45^{\circ },4000\;\text{Hz}\right)$, and the transmitting array elements emit an orthogonal phase code signal.

### 4.1. Simulation 1: The Restrained Ability for Spatial Gaussian Colored Noise in the Proposed Method

**Figure 1:**
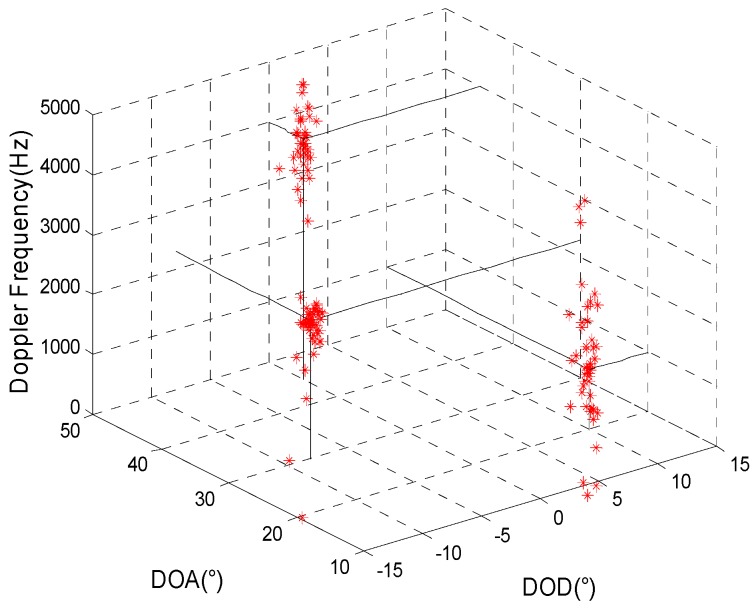
The estimation results of reference [8].

**Figure 2:**
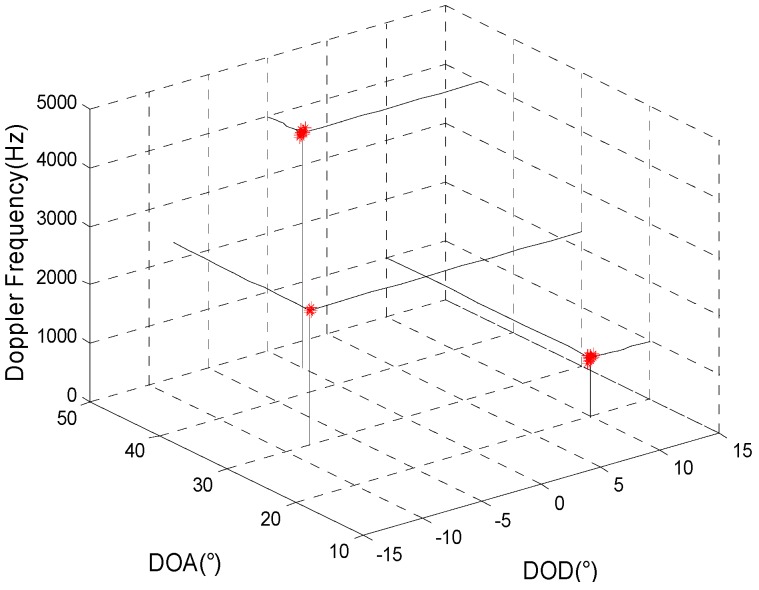
The estimation results of the proposed method.

The simulation mainly inspects the joint estimation result for target parameters in a background with Gaussian colored noise using the proposed method. It is assumed that the transmitting and receiving arrays are all uniform linear arrays and that the other simulation conditions are the same as in simulation 1. Figure 1 and Figure 2 present, respectively, the joint estimation results for target parameters using the algorithm in reference [8] and the method proposed here.

Figure 1 and Figure 2 show that a larger deviation of the estimation results for target parameters using the algorithm in reference [8] may occur as a result of the influence of spatial colored noise. For our proposed algorithm, the cross-correlation of the matched filters’ outputs for different moments is used so that the influence of spatial colored noise can be eliminated. Thus, the target parameters can be accurately estimated.

### 4.2. Simulation 2: When the Transmitting and Receiving Arrays are All Non-Uniform Linear Arrays, the Joint Estimation Results for the Target Parameters Using the Method Proposed here and the Algorithm Proposed in Reference [8]

**Figure 3:**
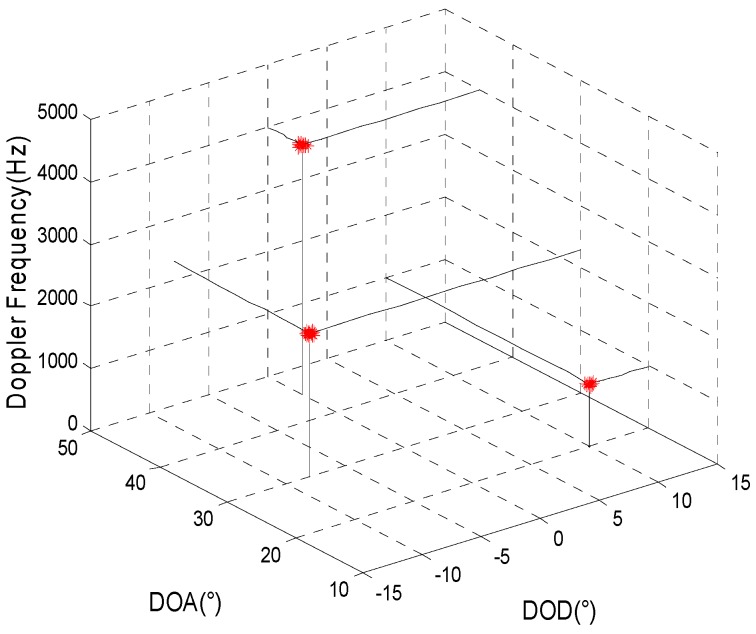
The estimation results of the proposed method for array set 1.

**Figure 4:**
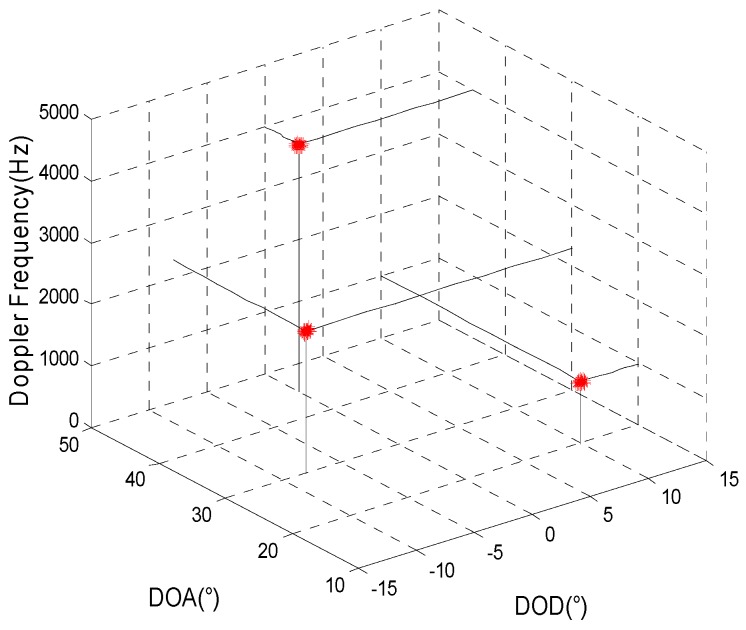
The estimation results of reference [13] for array set 1.

**Figure 5:**
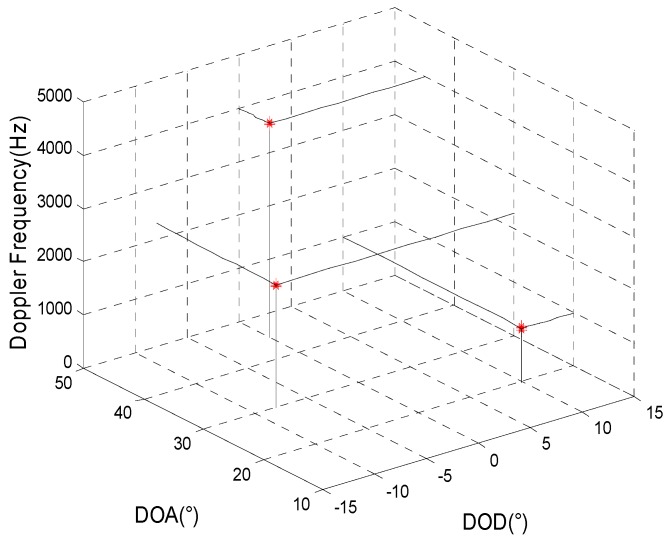
The estimation results of the proposed method for array set 2.

**Figure 6:**
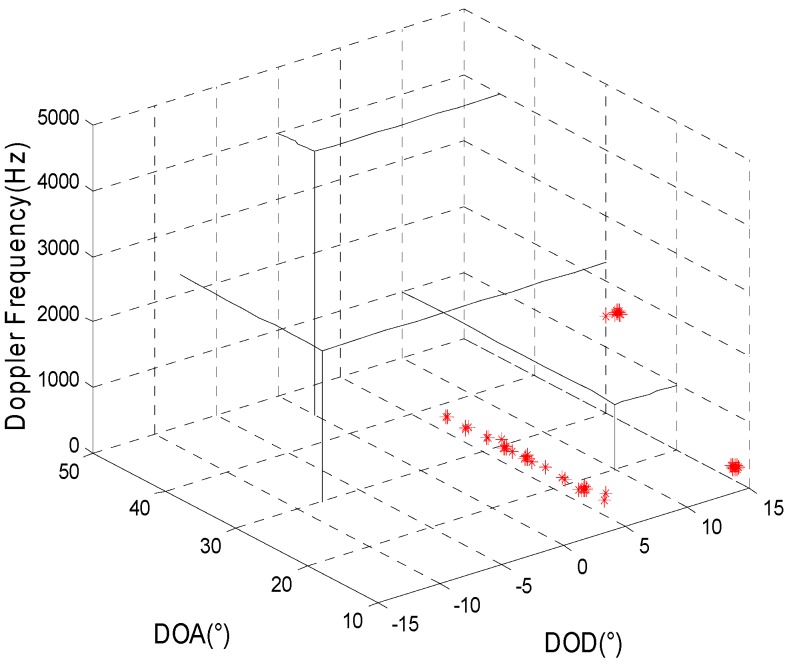
The estimation results of reference [13] for array set 2.

Comparing the joint estimation results for the target parameters obtained using the two algorithms for different non-uniform transmitting and receiving arrays, the simulation parameters are given as in Simulation 1. Figure 3 and Figure 4 show the joint estimation results for the target parameters using the method proposed here and the algorithm proposed in reference [13] when the array element positions of the transmitting and receiving arrays (called non-uniform array set 1) are, respectively, [0,0.5,0.92,1.38,1.83,2.31]×λ and [0,0.48,0.98,1.46,1.85,2.33,2.79,3.27]×λ. Figure 5 and Figure 6 show the joint estimation results for the target parameters obtained using the method proposed here and the algorithm proposed in reference [13] when the array element positions of the transmitting and receiving arrays (called non-uniform array set 2) are, respectively, [0,0.5,1,3,5,6.5]×λ and [0,0.5,2,5,8,9,10.5,11.4]×λ.

Shown as the simulation results when the positions of the transmitting and receiving arrays are set as [0,0.5,0.92,1.38,1.83,2.31]×λ and [0,0.48,0.98,1.46,1.85,2.33,2.79,3.27]×λ, respectively, which indicates that $d_{t,m}-d_{t,m-1}\leq \lambda /2,m=2,3,\cdots ,M_{t}$ and $d_{r,n}-d_{r,n-1}\leq \lambda /2,n=2,3,\cdots ,M_{r}$, the target parameters can be estimated accurately using the algorithm proposed in reference [13]. When the position of the transmitting and receiving arrays are set as [0,0.5,1,3,5,6.5]×λ and [0,0.5,2,5,8,9,10.5,11.4]×λ, respectively, which indicates that $d_{t,m}-d_{t,m-1}>\lambda /2,m=2,3,\cdots ,M_{t}$ and $d_{r,n}-d_{r,n-1}>\lambda /2,n=2,3,\cdots ,M_{r}$, the estimation results for the target parameters obtained using the algorithm in reference [13] may result in errors, which is consistent with the theoretical analysis in this paper. The target parameters can be accurately estimated regardless of the form of the array set using the method proposed in this paper. Therefore, the application of the method proposed here is not limited based on the form of the array.

### 4.3. Simulation 3: The Comparison Curve of the Statistical Performance of the Algorithm

**Figure 7:**
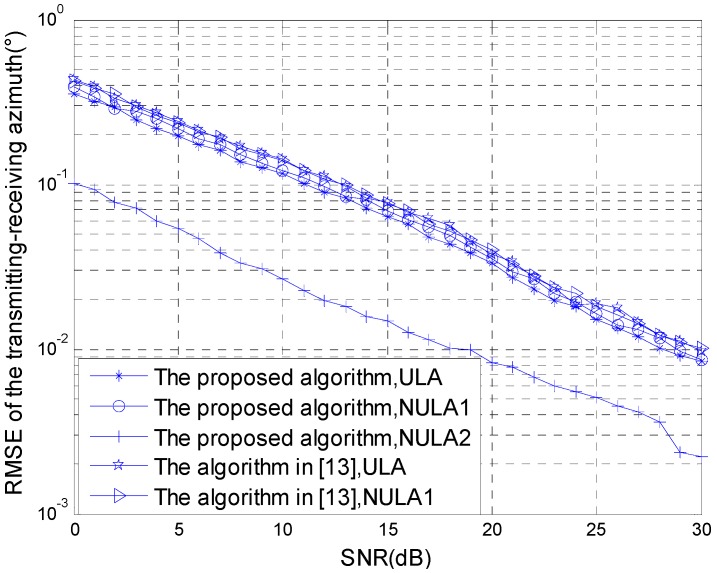
RMSE estimated for the transmitting-receiving azimuth.

**Figure 8:**
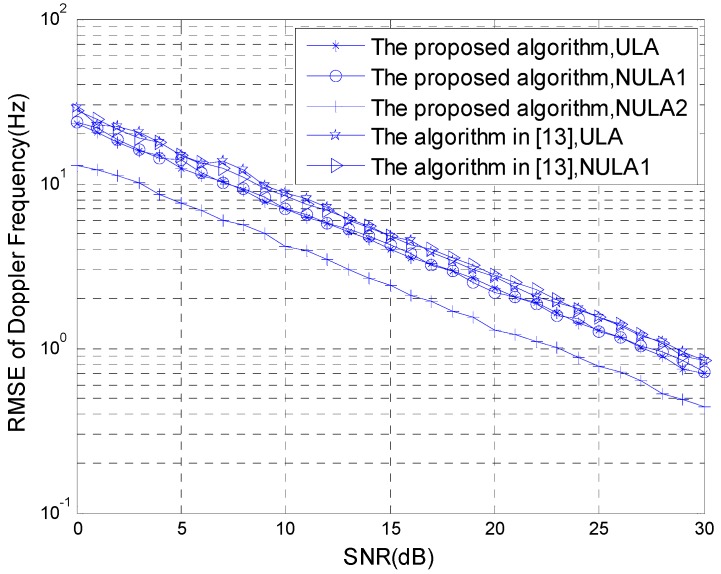
RMSE estimated for the Doppler frequency.

The statistical performance of the algorithms is compared with the SNR changes. The simulation parameters are given as in Simulation 2, and the simulation results were obtained using 200 Monte-Carlo experiments. Figure 7 and Figure 8 show, respectively, the comparison curve changed with the SNR of the RMSE estimated for the transmitting-receiving azimuth and the RMSE estimated for the Doppler frequency under different array set conditions.

According to the result of the Monte-Carlo experiment, if the non-uniform linear array set 2 is used in the transmitting and receiving arrays in the method proposed in this paper, the statistics estimation performance is superior. Although the algorithm in reference [13] is applicable to transmitting and receiving arrays that are non-uniform linear arrays, the distance between the transmitting and receiving arrays is not more than 0.5 times the wavelength. Thus, only the non-uniform linear array set 1 can be adopted. Therefore, for the method in reference [13], we can compare the transmitting and receiving arrays using a uniform linear array with the non-uniform linear array set 1, the statistics performance cannot be improved. The method with the non-uniform linear array set 2 proposed in this paper can greatly improve the statistics performance.

## 5. Conclusions

A spatio-temporal 2-D processing method for target parameter estimation for bistatic MIMO radar is proposed in this paper. In this method, the target DOD and DOA are first decoupled based on the diagonal loading method and are estimated. Then, the Doppler frequency is estimated based on the target 2-D azimuth estimation, thereby avoiding the multi-dimensional search of the joint parameters. Compared with the other existing estimation algorithms concerning the transmitting-receiving azimuth and Doppler frequency, the method proposed in this paper provides the following benefits:

(1) The prior estimate of the target number and the EVD of the data covariance matrix are not needed, thereby reducing the complexity and number of calculations.
(2) The estimated parameters can be automatically paired, and array aperture losses can be avoided.
(3) Compared with the algorithm in reference [13], the method proposed here does not include special demands on the structure of the transmitting and receiving arrays. The method is applied under a condition in which the transmitting and receiving arrays have an arbitrary geometrical configuration, and the method can greatly improve the parameter estimation performance. The algorithm in reference [13] can be used in transmitting and receiving arrays that do not satisfy conditions for translation invariant structure, but the distance between the two arbitrary elements of the transmitting and receiving arrays must not be more than 0.5 times the wavelength. Thus, the scope of the application of this method is limited to some degree.
(4) In the method proposed here, the outputs of the matched filters for different moments are cross-correlated to eliminate the spatial colored noise. Thus, this method is suitable for a wider background of colored noise.

To simplify the situation, we have only considered direct paths of reflected signals from a point target for MIMO radar in this paper. However, for low-angle estimation, multipath and the deleterious effects of the atmosphere [14,15] play a large role in degrading the accuracy of angle estimation algorithms. Therefore, angle estimation for bistatic MIMO radar that addresses multipath and the deleterious effects of the atmosphere will be researched in the future.
